# Chronic administration of parecoxib exerts anxiolytic-like and memory enhancing effects and modulates synaptophysin expression in mice

**DOI:** 10.1186/s12871-017-0443-y

**Published:** 2017-11-13

**Authors:** Bo Wang, Xin Jin, Xin Kuang, Shaowen Tian

**Affiliations:** 1 Department of Anesthesiology, First Affiliated Hospital, University of South China, Hengyang, Hunan 421001 People’s Republic of China; 2 Department of Anesthesiology, Nanhua Affiliated Hospital, University of South China, Hengyang, Hunan 421001 People’s Republic of China; 3 Department of Physiology, College of Medicine, University of South China, Hengyang, Hunan 421001 People’s Republic of China

**Keywords:** Cyclooxygenase, Parecoxib, Anxiety, Memory, Synaptophysin

## Abstract

**Background:**

Previous studies have shown that cyclooxygenase-2, a key enzyme that converts arachidonic acid to prostaglandins, is involved in anxiety and cognitive processes, but few studies have investigated the effects of chronic administration of cyclooxygenase-2 inhibitors on anxiety, learning and memory under normal physiological conditions. The aim of the study was to investigate the effects of chronic administration of parecoxib, a cyclooxygenase-2 inhibitor, on anxiety behavior and memory performance under normal physiological conditions and to explore the possible neural mechanism underlying parecoxib-mediated effects.

**Methods:**

Adult male ICR mice were randomly divided into four groups: the control group and three parecoxib groups. Mice received normal saline or parecoxib (2.5, 5.0 or 10 mg/kg) intraperitoneal injection once a day for 21 days, respectively. Elevated plus-maze, novel object recognition and Y maze tests were conducted on day 23, 24 and 26, respectively. Four additional groups that received same drug treatment were used to measure synaptophysin protein levels by western blot and prostaglandin E2 (PGE2) levels by ELISA in the amygdala and hippocampus on day 26.

**Results:**

Chronic parecoxib exerted an anxiolytic-like effect in the plus-maze test test, and enhanced memory performance in the novel object recognition and Y maze tests. Western blot analysis showed that chronic parecoxib down-regulated synaptophysin levels in the amygdala and up-regulated synaptophysin levels in the hippocampus. ELISA assay showed that chronic parecoxib inhibited PGE2 in the hippocampus but not amygdala.

**Conclusions:**

Chronic parecoxib exerts anxiolytic-like and memory enhancing effects, which might be mediated through differential modulation of synaptophysin and PGE2 in the amygdala and hippocampus.

## Background

Cyclooxygenase is a key enzyme that converts arachidonic acid to prostaglandins. Cyclooxygenase comes in two isoforms: cyclooxygenase-1 and cyclooxygenase-2. In the brain, cyclooxygenase-2 is expressed in neurons in both physiological and pathological processes. It is enriched in brain regions that are critically involved in anxiety behavior and cognitive function [1, 2], such as the amygdala and hippocampus [3]. Within neurons, cyclooxygenase-2 immunoreactivity is localized in dendritic spines [3, 4], which are specialized structures involved in anxiety behavior and synaptic plasticity [5, 6].

Recent studies have suggested that cyclooxygenase-2 is involved in anxiety behavior. Mice with traumatic brain injury show increased anxiety-like behavior and cyclooxygenase-2 expression [7]. Stress results in heightened anxiety-like behavior and increased levels of cyclooxygenase-2 in the hippocampus of rats [8]. Sleep deprivation produces an increase in anxiety-like behavior and cyclooxygenase-2 expression [9], while substrate-selective inhibition of cyclooxygenase-2 reduces anxiety-like behavior in mice [10]. cyclooxygenase-2 inhibitors improve stress-induced anxiety-like behavior in mice [11]. A clinical study showed that preventative administration of cyclooxygenase-2 inhibitor parecoxib exerts anxiolytic effects in patients undergoing total knee arthroplasty [12]. However, comparatively few studies have investigated the effects of cyclooxygenase-2 inhibitors on anxiety-like behavior under normal physiological conditions.

A considerable body of evidence indicates that cyclooxygenase-2 plays an important role in learning and memory. Under normal physiological conditions, cyclooxygenase-2 inhibitors impair memory acquisition and/or retention in the water maze task in rats and in the one-trial passive avoidance task in chicks [13, 14]. Under pathological conditions, cyclooxygenase-2 inhibitors can improve memory performance in the Morris water maze, passive avoidance, elevated plus maze, radial arm water maze and in contextual fear conditioning tasks [15–17]. At the same time, some observations indicate that cyclooxygenase-2 inhibitors fail to modulate normal memory performance in the Morris water maze, passive avoidance, elevated plus maze, and object-place recognition tasks [16, 18]. At present, however, the effects of cyclooxygenase-2 inhibitors on memory performance in the novel object recognition and Y-maze tasks need to be elucidated.

Synaptophysin is a membrane protein in synaptic vesicles, and is used as a specific marker of synaptic vesicles/synaptic terminals [19]. This protein plays an important role in neurotransmitter release and the synaptic vesicle cycle. Recent studies have suggested that synaptophysin is involved in anxiety behavior and cognitive process [20, 21]. A previous study indicates that cyclooxygenase-2 inhibition modulates synaptophysin expression in metastatic prostatic cancer cells [22]. However, the role of synaptophysin in cyclooxygenase-2 inhibition-mediated effects needs to be determined.

We investigated the effects of chronic administration of parecoxib on anxiety behavior in the elevated plus-maze test, and on memory performance in the novel object recognition and Y maze tests under normal physiological conditions in mice. In addition, synaptophysin protein and PGE2 levels in the amygdala and hippocampus was measured, to explore the possible underlying mechanisms.

## Methods

### Experimental animals

We used naive adult male ICR mice, 15 weeks old and weighing 25–35 g, obtained from the Hunan SJA Laboratory Animal Co.,Ltd. (Changsha, China), for this experiment. The animals were housed in groups of 4–5 per cage in a temperature- and humidity-controlled environment, with ad libitum access to food and water. They were maintained on a 12 h light/dark schedule, and lights were switched on at 7 A.M. The animals were handled (5–6 min per animal per day) for 1 week, after being housed, to habituate them to the experimenter. Experiments were conducted according to the *National Institutes of Health Guide for the Care and Use of Laboratory Animals*, and the University of South China Animal Care and Use Committee approved experimental protocols (Animal study approval number: SYXK(Xiang)2010–0006).

### Drug administration

**Fig. 1:**
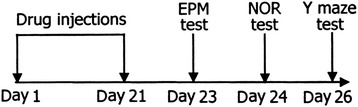
Schematic of the experimental designs. Mice were given intraperitoneal injections of parecoxib sodium (2.5, 5.0 or 10 mg/kg) for 21 days. Elevated plus-maze (EPM), novel object recognition (NOR) and Y maze tests were conducted on day 23, 24 and 26, respectively

The mice were randomly divided into four groups: the control group (*n* = 10) and three parecoxib groups (*n* = 9 per group). Parecoxib sodium (2.5, 5.0 or 10 mg/kg; Dynastat, Pfizer, NY, USA) was injected intraperitoneally into the mice, once a day for 21 days. The doses were chosen according to a pharmacological profile study of parecoxib [23]. The control mice were treated similarly, except that normal saline was used. Behaviour tests (Elevated plus-maze, novel object recognition and Y-maze tests) were conducted at corresponding time points as indicated in Fig. 1.

Four additional groups were used to measure synaptophysin protein (*n* = 4 per group) and prostaglandin E2 (PGE2) (*n* = 5 per group) levels in the amygdala and hippocampus. The control and parecoxib groups received saline or parecoxib (2.5, 5 and 10 mg/kg) injection once a day for 21 days as described above. To avoid the potential effect of behavioral tests on synaptophysin protein expression and prostaglandin E2, the four groups were not submitted to behavioral tests. The mice were anesthetized and sacrificed by decapitation 5 days after the cessation of drug administration. The time point corresponded to the end of the behavioral tests. The amygdala and hippocampus were quickly removed from the skull and then extracted. The tissues were stored at −80 °C until use.

### Elevated plus-maze test

The elevated plus-maze test is a proven model used to assess the level of anxiety in rodents. Details of the apparatus and the procedure were described in our previous study [24]. Briefly, the mice were taken from their cages and placed in the apparatus for 5 minutes. The parameters observed were the number of entries into the open and closed arms, and time spent in the open arms. The first and the last parameters were expressed as a percentage. The number of entries into the closed arms was considered as the locomotor activity index. The percentage of the time spent in the open arms and the percentage of the open arms entries were considered the anxiety index [25, 26]. To avoid the potential effects of the last injection of parecoxib, the elevated plus-maze test was conducted 2 days after the cessation of drug administration.

### Novel object recognition test

Details of the apparatus and the procedure were described in our previous study [27]. However, we used smaller objects (4 × 4 × 6 cm) in this study. The behavioral procedure involved two sessions: sampling and retention test with an interval of twenty-four hours. In the sampling session, the mouse was placed in the training apparatus and allowed to explore two identical objects for 5 minutes. The total time spent exploring both objects was recorded. Exploration of an object was defined as pointing the nose toward the object at a distance of <1 cm and/or touching it with the nose. In the retention test session, one copy of the familiar object and a new object were placed in the same location as stimuli in the sampling session. The mouse was placed in the training apparatus for 5 minutes. The times spent exploring each object and the total time spent exploring both objects were recorded. The discrimination index used to assess memory performance was calculated as the difference in exploration times between the novel and familiar objects divided by the total time spent exploring both the objects. The novel object recognition test was started 3 days after the cessation of drug administration.

### Y-maze test

The apparatus consisted of a Y-maze with three arms (30 cm × 8 cm), surrounded by black Plexiglass walls (15 cm high). The central platform of triangular shape measured 8 × 8 × 8 cm. The Y-maze test was conducted as previously described [28]. Briefly, each mouse was placed at the end of one of the arms (facing the end of the arm), and was allowed to explore all the arms freely during an 8 minutes testing period. We recorded the series of entries and the total number of entries. An entry into an arm was defined as the animal placing all four paws inside that arm. An alternation was defined as visits into all three arms on consecutive occasions. The number of maximum alternations was the total number of arm entries minus two. The alternation ratio was defined as the number of alternations divided by maximum alternations. The total number of arm entries was considered as the locomotor activity index and the alternation ratio as the memory index. This test was conducted 5 days after the cessation of drug administration.

### Detection of synaptophysin protein expression in the amygdala and hippocampus by western blot

Tissues were homogenized in RIPA Lysis Buffer (Beyotime Institute of Biotechnology, Haimen, Jiangsu, China). They were centrifuged at 12000 rpm/min for 6 minutes. We collected the supernatant and kept it at −70 °C. Protein content was further determined with the BCA assay kit (Beyotime Institute of Biotechnology, Haimen, Jiangsu, China). Sample proteins were separated by sodium dodecyl sulfate polyacrylamide gel electropheresis, and then transferred to polyvinylidene fluoride membranes (Sigma Chemical Co., St. Louis, MO, USA). The membranes were blocked for 60 min in Tris-buffered saline containing 0.05% Tween-20 and 5% bovine serum albumin. The membranes were then incubated with mouse anti-synaptophysin antibody (#17785–1-AP, Proteintech, USA) or with mouse anti-β-actin antibody (#60008–1-Ig, Proteintech, USA) and shaken for 1 hour at 37 °C. The membranes were then washed and incubated with secondary antibody (Goat Anti-Rabbit IgG/HRP) and shaken for 45 min at 37 °C. The membranes were washed and the specific proteins were then visualized using an enhanced chemiluminescence detection kit (Beyotime Institute of Biotechnology, Haimen, Jiangsu, China). The intensity of protein bands of synaptophysin and β-actin were quantified by densitometry.

### Detection of PGE2 in the amygdala and hippocampus by ELISA

As previously described [29], PGE2 levels in the amygdala and hippocampus were measured using a PGE2 ELISA kit (ab133021, Abcam, USA) according to the manufacturer’s protocol. The PGE2 levels were expressed as ng/mg of protein.

### Statistical analyses

Statistical analyses were performed using unpaired *t*-test or one-way analysis of variance (ANOVA) (Sigma Stat 3.2). *Post-hoc* comparisons were performed with the Tukey HSD method. In addition, one-sample *t*-test was used to compare the discrimination index of the control group with chance performance (discrimination index = 0%). The data were represented as mean ± SEM. The significance level was set at *P* < 0.05.

## Results

### Chronic administration of parecoxib exerts an anxiolytic-like effect in the elevated plus-maze test

**Fig. 2:**
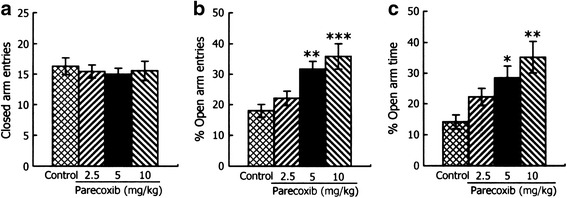
Effects of chronic administration of parecoxib on anxiety behavior in the elevated plus-maze test. The number of the closed arms entries (**a**), the percentage of the open arms entries (**b**) and the percentage of time spent in the open arms (**c**) in the elevated plus-maze test. *P < 0.05, **P < 0.01 and ***P < 0.001 versus the control group. All data are represented as mean ± SEM

The effects of chronic administration of parecoxib in the elevated plus-maze test are shown in Fig. 2. For the number of closed arms entries (Fig. 2a), a one-way ANOVA revealed no significant difference among the four groups (*F*_(3,33)_ = 0.184, *P* > 0.05), indicating that chronic parecoxib administration does not affect locomotor activity. For the percentage of the open arms entries (Fig. 2b), a one-way ANOVA revealed a significant difference among the four groups (F_(3, 33)_ = 8.454, *P* < 0.001). *Post-hoc* comparisons showed that when compared with the control group, mice treated with parecoxib at 5 and 10 mg/kg showed higher levels of open arms entry percentages (*p* < 0.01, *P* < 0.001, respectively). For the percentage of time spent in the open arms (Fig. 2c), a one-way ANOVA revealed a significant difference among the four groups (F_(3,33)_ = 6.278, *P* < 0.01). Post hoc comparisons showed that when compared with the control group, mice treated with parecoxib at 5 and 10 mg/kg showed higher levels of percentage of time spent in the open arms (*P* < 0.05 and *P* < 0.01, respectively). These results suggest that chronic administration of parecoxib exerts an anxiolytic-like effect in the elevated plus-maze test.

### Chronic administration of parecoxib enhances memory performance in the novel object recognition test

**Fig. 3:**
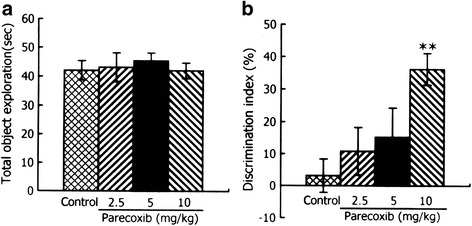
Effects of chronic administration of parecoxib on memory performance in the novel object recognition tests. The total time spent exploring both objects in the sampling session (**a**) and the discrimination index in the retention test session (**b**) in the novel object recognition test. **P < 0.01 versus the control group. All data are represented as mean ± SEM

A one-way ANOVA revealed no significant difference in the total time spent exploring both objects between the four groups in the 5 minutes sampling session (F_(3, 33)_ = 0.181, *P* > 0.05) (Fig. 3a). In the 5-min retention test session, one-sample *t*-tests indicated that the control group exhibited a chance performance (*P* > 0.05). A one-way ANOVA revealed a significant difference in the discrimination index between the four groups (F_(3, 33)_ = 4.358, *P* < 0.05) (Fig. 3b). *Post-hoc* comparisons showed that when compared with the control group, mice treated with parecoxib at 10 mg/kg showed a significant increase of discrimination index (*P* < 0.01). There was no significant difference in the discrimination index between the control mice and those treated with parecoxib at 2.5 or 5 mg/kg (both *P* > 0.05). These results suggest that chronic administration of parecoxib enhances memory performance in the novel object recognition test.

### Chronic administration of parecoxib enhances memory performance in the Y-maze test

**Fig. 4:**
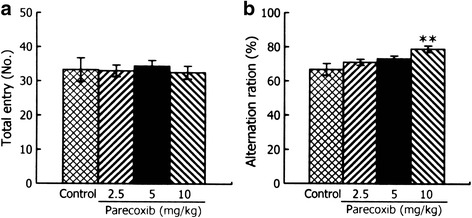
Effects of chronic administration of parecoxib on memory performance in the Y-maze task. The number of total entries (**a**) and alternation ratio (**b**) in the Y-maze task. **P < 0.01 versus the control group. All data are represented as mean ± SEM

There was no significant main effect of groups in the number of total entries (F_(3,33)_ = 0.111, *P* > 0.05) (Fig. 4a), indicating that chronic parecoxib administration does not affect locomotor activity. For the alternation ratio, a one-way ANOVA revealed a significant difference between the four groups (F_(3,33)_ = 4.054, *P* < 0.05) (Fig. 4b). *Post-hoc* comparisons showed that when compared with the control group, mice with parecoxib at 10 mg/kg showed significant increase in the alternation ratio (*P* < 0.01). There was no significant difference in the alternation ratio between the control mice and the mice treated with parecoxib, at doses of 2.5 and 5 mg/kg (both, *P* > 0.05). These results suggest that chronic administration of parecoxib enhances memory performance in the Y-maze test.

### Chronic administration of parecoxib modulates synaptophysin protein expression in the amygdala and hippocampus

**Fig. 5:**
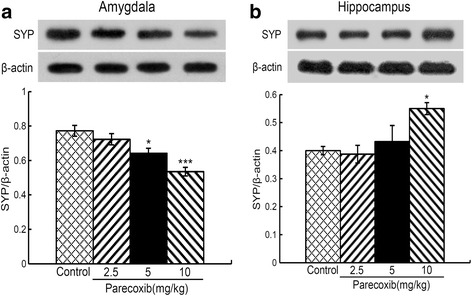
Effects of chronic administration of parecoxib on synaptophysin protein expression in the amygdala and hippocampus. The protein band (top) and the relative expression levels of synaptophysin (down) in the amygdala (**a**) and hippocampus (**b**). *P < 0.05 and ***P < 0.001 versus the control group. SYP, synaptophysin; All data are represented as mean ± SEM

The effects of chronic administration of parecoxib on synaptophysin protein expression in the amygdala and hippocampus are shown in Fig. 5. In the amygdala, a one-way ANOVA revealed a significant difference in the synaptophysin protein levels between the four groups (F_(3, 12)_ = 12.162, *P* < 0.001) (Fig. 5a). *Post-hoc* comparisons showed that when compared with the control group, mice treated with parecoxib at 5 and 10 mg/kg showed a significant decrease in the levels of synaptophysin protein expression (*P* < 0.05 and *P* < 0.001, respectively). In the hippocampus, a one-way ANOVA revealed a significant difference in the synaptophysin protein levels between the four groups (F_(3, 12)_ = 4.445, *P* < 0.05) (Fig. 5b). *Post-hoc* comparisons showed that when compared with the control group, only mice treated with parecoxib at 10 mg/kg showed a significant increase in the levels of synaptophysin protein expression (*P* < 0.05).

### Chronic administration of parecoxib inhibits PGE2 in the hippocampus but not amygdala

**Fig. 6:**
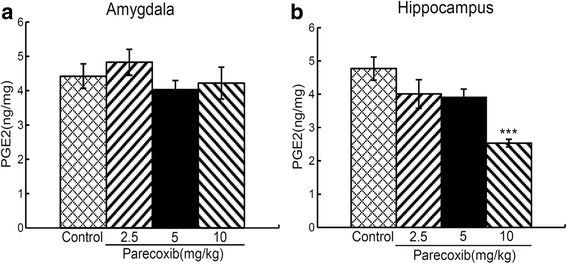
Effects of chronic administration of parecoxib on PGE2 in the amygdala and hippocampus. The PGE2 levels (ng/mg) in the amygdala (**a**) and hippocampus (**b**). ***P < 0.001 versus the control group. All data are represented as mean ± SEM

The effects of chronic administration of parecoxib on PGE2 in the amygdala and hippocampus are shown in Fig. 6. In the amygdala, a one-way ANOVA revealed no significant difference in the PGE2 levels between the four groups (F_(3, 16)_ = 0.845, *P* > 0.05) (Fig. 6a). In the hippocampus, a one-way ANOVA revealed a significant difference in the PGE2 levels between the four groups (F_(3, 16)_ = 9.149, *P* < 0.001) (Fig. 6b). *Post-hoc* comparisons showed that when compared with the control group, only mice treated with parecoxib at 10 mg/kg showed a significant decrease in the levels of PGE2 (*P* < 0.001).

## Discussion

In this study, we first investigated the effects of chronic administration of cyclooxygenase-2 inhibitor parecoxib on anxiety behavior in the elevated plus-maze test, and on memory performance in the novel object recognition and Y maze tests in mice. The results indicate that chronic administration of parecoxib exerts anxiolytic-like and memory enhancing effects under normal physiological conditions. It could be argued that the non-cognitive components, such as motor stimulant effects or anxiolytic-like activity, could be confounding factors in the evaluation of memory performance of the animals. Indeed, our current results found that chronic parecoxib administration did not affect locomotor activity as indicated by the number of the closed arms entries in the elevated plus-maze test and the number of total entries in the Y-maze test. Furthermore, the novel object recognition and Y-maze tasks require little training of animals and do not induce high levels of stress and arousal [30]. Thus, the motor stimulant effects or anxiolytic-like activity of chronic parecoxib administration are unlikely to be involved in these memory enhancing effects.

Our finding is in accordance with previous observations that cyclooxygenase-2 inhibition reduces anxiety-like behavior [10–12]. The specific mechanism through which chronic parecoxib administration exerts an anxiolytic-like effect remains unclear. Recent researches have suggested that synaptophysin is involved in anxiety behavior. For example, mice with traumatic brain injury show increased anxiety-like behavior and decreased levels of synaptophysin in the hippocampus. Such effects can be reversed by the fatty acid amide hydrolase inhibitor PF-3845 [7]. Physical exercise improves anxiety-like behavior and restores down-regulation of synaptophysin in the hippocampus of 3xTg-AD mice [31]. Rats with neonatal hypoxia-ischemia or maternally separation (MS180min) cause increased levels of anxiety and decreased levels of synaptophysin in the hippocampus [32]. In a model of complex regional pain syndrome, fracture/cast mice show signs of anxiety and reduction of synaptophysin levels in the hippocampus [33]. Chronic central administration of ghrelin produces an increase in anxiety-like behavior and synaptophysin gene expression in the amygdala in rats [34]. Predator threat stress promotes long lasting anxiety-like behavior and up-regulates synaptophysin gene expression in the amygdala in rats [35]. The current results indicate that chronic parecoxib administration decreases synaptophysin levels in the amygdala and increases synaptophysin levels in the hippocampus. This bi-directional modulation of synaptophysin protein expression in the amygdala and hippocampus might mediate the anxiolytic-like effect of chronic parecoxib administration.

The specific mechanism underlying chronic parecoxib-induced bi-directional change of synaptophysin in the amygdala and hippocampus is unclear. COX-2 and the PGE2 subtype 2 receptor are colocalized with synaptophysin in presynaptic terminals [36]. Cyclooxygenase-2 inhibition increases expression of synaptophysin in metastatic prostate cancer cells [22]. The current results show that chronic parecoxib inhibits cyclooxygenase-2 activity as indicated by a decrease of the PGE2 levels, which may lead to up-regulation of synaptophysin in the hippocampus. However, the current available data is difficult to explain the effect of chronic parecoxib on synaptophysin in the amygdala. In fact, chronic parecoxib has no effect on the PGE2 levels in this region, suggesting that down-regulation of synaptophysin is not due to cyclooxygenase-2 inhibition. Future research is needed to elucidate this issue.

Previous studies have suggested that cyclooxygenase-2 is involved in learning and memory. In some of the investigations, administration of cyclooxygenase-2 inhibitors caused impairment in memory performance in rats [13, 14]. Our results do not support the idea that cyclooxygenase-2 inhibition impairs memory. In fact, our findings are in accordance with the suggestion that cyclooxygenase-2 inhibition can improve memory in rats or mice [15, 16]. This controversy might be due to the nature of the task, the kind of cyclooxygenase-2 inhibitor used and its administration schedule, and the species and ages of animals used.

Previous researches have suggested that synaptophysin is implicated in learning and memory. For example, heat stress-induced memory impairment in the novel object recognition and Y-maze tasks is associated with a decreased level of synaptophysin expression in the hippocampus [37]. Sitagliptin, a dipeptidyl peptidase-4 inhibitor, improves novel object recognition memory and increases gene expression of hippocampal synaptophysin [38]. In a model of Alzheimer’s disease, Aldh2 (−/−) mice showed age-related memory deficits in the novel object recognition and Y-maze tasks and a decrease of hippocampal synaptophysin levels [39]. Heat stress induces an increase in cyclooxygenase-2 and a decrease in synaptophysin in the hippocampus [37]. RNA interference-mediated cyclooxygenase-2 inhibition enhances expression of synaptophysin [22]. Consistent with these studies, our study demonstrates that cyclooxygenase-2 inhibition by chronic parecoxib administration increases synaptophysin expression in the hippocampus. Possibly, chronic parecoxib-induced increase of synaptophysin levels in the hippocampus might contribute to the memory facilitating effects in the novel object recognition and Y-maze tasks.

## Conclusion

In conclusion, chronic administration of parecoxib exerts anxiolytic-like and memory enhancing effects under normal physiological conditions in mice. These effects might be mediated through differential modulation of synaptophysin expression in the amygdala and hippocampus. Our finding extends the researches on the effects of cyclooxygenase-2 inhibitors on anxiety, learning and memory.

## Abbreviations

ELISA: Enzyme-linked immunosorbent assay
PGE2: Prostaglandin E2
